# Patterns of information flow in local cortical networks

**DOI:** 10.1186/1471-2202-15-S1-P213

**Published:** 2014-07-21

**Authors:** Sunny Nigam, Olaf Sporns, Masanori Shimono, John M Beggs

**Affiliations:** 1 Department of Physics, Indiana University, Bloomington, IN 47405, USA; 2 Department of Psychological and Brain Sciences, Indiana University, Bloomington, IN 47405, USA

## 

**Figure 1:**
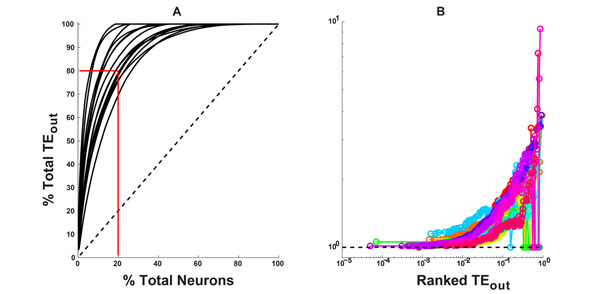
**A**) Cumulative distribution of total-outgoing TE (solid black lines) as a function of the percent of contributing neurons. Dotted black line shows what the distribution should have been for a network with uniform distribution of information flow. **B**) Normalized weighted rich club coefficient plotted versus the ranked richness parameter (TE_out_). A coefficient greater than 1 for a range of values of the ranked richness parameter implies the existence of a rich club in the network

Structural and functional connectivity of macroscopic brain regions has been very widely researched [1] in the last decade, however very little work has been done on the effective connectivity between individual neurons, largely because of limitations on the simultaneous measurement of large numbers of neurons at high spatiotemporal resolution. We recorded spontaneous single neuron activity from 15 organotypic cultures (prepared from the mouse cortex), with a 512 channel micro-electrode array at a temporal resolution of less than 1 ms and a spatial resolution of 60 µm. The average number of recorded neurons was 347 ± 119. Effective connectivity matrices, both binary and weighted, were constructed from the spike trains using transfer entropy (TE) analysis to estimate directed neuronal interactions [2]. The strength of information flow from neuron *i* to *j* was quantified in terms of the TE value calculated from neuron *i* to *j*. We observed that only 20% of the recorded neurons accounted for 80% of the total information flow in these networks (see Figure 1A) which we define as the network’s set of *rich* nodes. The rich nodes were characterized by a higher firing rate, and graph theoretic analysis revealed their participation in a number of highly non-random network features. The networks were highly clustered with small average path lengths and thus exhibited small world attributes and the sub-network formed by the rich nodes was more efficient than the rest of the network. The networks also exhibited a central core of rich nodes which persisted even when the rest of the nodes in the network were peeled away. The rich nodes were also connected to each other more strongly than expected by chance giving rise to the *rich club* effect, observed for the first time in effective connectivity networks of individual neurons (see Figure 1B). The rich club effect has previously been shown to exist in the structural connectivity between macroscopic brain regions in cat, macaque monkey and humans [3] and between neurons in C. *Elegans *[4].Our findings suggest that networks of TE effective connectivity in mouse cortex contain a subset of highly connected and highly interactive neurons reminiscent of rich club organization observed at larger scales.
